# Analysis and engineering of quorum sensing-based communications between bacteria and fungi

**DOI:** 10.1128/mbio.03838-25

**Published:** 2026-03-09

**Authors:** Yongsheng Zhou, Lingjin Tang, Danlei Chen, Yanni Li, Shengbo Wu, Jianjun Qiao

**Affiliations:** 1 School of Synthetic Biology and Biomanufacturing, Key Laboratory of Systems Bioengineering (Ministry of Education), State Key Laboratory of Synthetic Biology, Tianjin University 12605, Tianjin, China; 2 Zhejiang Institute of Tianjin University (Shaoxing), Shaoxing, China; Vallabhbhai Patel Chest Institute, Delhi, India

**Keywords:** quorum sensing, bacterial-fungal interactions, cross-kingdom communication, synthetic biology, microbial community, cell-cell signal modification

## Abstract

Bacteria and fungi ubiquitously coexist, with their interactions critically influencing human health and industrial processes. Quorum sensing (QS) is a core regulatory mechanism that enables density-dependent coordination and phenotypic responses across these two kingdoms. While bacteria and fungi utilize their respective QS systems to engage in competitive or cooperative interactions to enhance their environmental adaptability, the current understanding of QS-based communications between them remains scattered, and a systematic summary of this field is still lacking. In this review, we examine the intricate dialog between bacteria and fungi, focusing on its role in microbial network assembly and ecosystem function, to provide a comprehensive analysis and engineering perspective on QS-based cross-kingdom communication. Specifically, we will first briefly delineate the core architecture of bacterial and fungal QS systems and the phenotypes they govern. Then, we will analyze QS-based interactions across diverse environments between different bacteria and fungi, categorizing natural QS interactions based on various phenotypes, including biofilm co-assembly and metabolic complementation. We further compare and analyze synthetic biology strategies, including promoter engineering and directed evolution of QS regulatory components, for reprogramming bacterial-fungal interactions and their applications. By synthesizing and contrasting these natural paradigms with synthetic designs, we provide a blueprint for achieving modular control over bacterial-fungal communities in diverse environments. Finally, by outlining persistent challenges and future trends, we aim to propel this field forward, enabling the deciphering of complex microbial interactions and ultimately increasing our capacity to engineer microbial consortia for diverse applications.

## INTRODUCTION

In natural environments, bacteria and fungi nearly always coexist, and their interactions play pivotal roles in maintaining human health (1, 2) and driving industrial processes (3). In the gut, for instance, bacterial communities facilitate nutrient absorption (4), whereas fungi, through resource competition, help prevent pathogenic bacteria from overgrowing (5, 6). Disruption of this balance is associated with various diseases, including enteritis and systemic fungal infections (7). In industrial applications, cooperative interactions between bacteria and fungi can enhance production efficiency and output quality (8). Fungi can produce cellulase to decompose raw materials, and bacteria can convert substrates into products such as ethanol (9) and antibiotics. Therefore, the analysis and engineering of the interactions between bacteria and fungi are essential for understanding microbial interplay and advancing the applications of microbial communities.

Quorum sensing (QS) serves as a core regulatory mechanism in bacterial-fungal interactions, enabling density-dependent coordination and phenotypic control through diffusible signaling molecules. Phenotypically, QS regulates key processes such as biofilm formation (10), metabolic complementation (11), and virulence expression (12, 13). These interactions occur naturally across diverse environments (14–16), and engineered QS systems allow precise control over interaction dynamics (17, 18), offering novel strategies for diverse applications. Deciphering QS-based bacterial-fungal communications is essential for understanding microbial network assembly and ecosystem function (19–22). In synthetic ecosystems, QS supports the division of labor and coordinated population behaviors, providing a blueprint for designing robust, functional microbial communities (23). With the rise of fungal research (24, 25), advances in QS-based bacterial-fungal interactions thus offer a framework for unraveling microbial social networks and bridging fundamental insights with applications in environmental and biomedical fields.

Nevertheless, current understanding of QS-based communications between bacteria and fungi remains scattered, and a systematic summary of this field is still lacking. A systematic analysis of QS-based communication and its resulting ecological impacts within natural bacterial-fungal communities will facilitate a deeper understanding of how these microorganisms coordinate collective behaviors and adapt physiologically in varied microenvironments. Furthermore, engineering controllable bacterial-fungal interactions is crucial for advancing their applications, highlighting the need to optimize genetic circuitry and regulatory control in synthetic coculture systems.

In this review, we will explore the dialog between bacteria and fungi. We will begin by delineating the core architecture of bacterial and fungal QS systems and the phenotypes they govern. Then, we will conduct the analysis and engineering of QS-based communications between bacteria and fungi. With respect to the former, we will analyze QS-based interactions between different bacteria and fungi, categorizing natural QS interactions based on various phenotypes, including biofilm co-assembly, metabolic complementation, and other functions. With regard to the latter, we will highlight the engineering of bacterial-fungal interactions, which are primarily focused on modifications of promoters and other QS regulatory elements. Finally, we will discuss the current challenges and opportunities for the further development of QS-based ecological interactions. We uniquely highlight the translation of natural QS circuitry into controllable genetic components for synthetic cocultures, providing a strategic roadmap for engineering stable, high-performance microbial consortia.

## FUNDAMENTALS OF BACTERIAL AND FUNGAL QS MECHANISMS

QS is a molecular mechanism that enables cell-to-cell communications mediated by diverse signaling molecules (QSMs). In bacteria, QSMs such as N-acyl-homoserine lactones (AHLs) and autoinducing peptides (AIPs) trigger cascade reactions that orchestrate gene expression and collective behaviors (26–29). In contrast, fungi typically employ aromatic alcohols, pheromones, and other compounds to regulate processes including virulence, morphological switching, and biofilm formation (30). The following sections will outline the core QS mechanisms and key regulated phenotypes in bacteria and fungi, respectively (Table 1). For more comprehensive discussions on the specific architectures of QS systems, the readers are referred to specialized reviews (30–35).

**TABLE 1:** Common QS systems of bacteria and fungi and their regulatory phenotypes^*a*^

Microbes	QS systems	Regulated phenotypes	Reference
Bacteria			
*Escherichia coli*	SdiA	Acid resistance, colonization and survival, drug resistance, motility, adherence, and biofilm formation	(27)
*Sphingomonas rubra*	C_4_-HSL, C_6_-HSL, C_10_-HSL, C_14_-HSL	Nitrification and denitrification, particle aggregation, and biofilm formation	(36)
*Pseudomonas aeruginosa*	3-O-C_12_-HSL, C_4_-HSL	Changes in the population density and species composition of the vicinal community	(28)
*Burkholderia thailandensis*	3-OH-C_8_-HSL,3-OH-C_10_-HSL	Regulate the production of antimicrobials and proteases	(37)
*Escherichia coli*	AI-2/ AI-3	Surface colonization and adherence, chemoreception and aggregation, biofilm and motility, drug resistance	(27)
*Lactococcus lactis*	NisinA	Biofilm formation, acid stress tolerance, bacteriocin production, morphological switches, and oriented growth	(38)
*Salmonella typhimurium*	AI-2	Virulence and biofilm formation	(39)
*Bacillus subtilis*	comX	Cell communication and gene regulation	(40)
*Streptococcus mutans*	CSP	Regulating biofilm formation and expression of virulence factors	(40)
*Xanthomonas campestris*	DSF	Virulence and biofilm formation	(41)
*Pseudomonas aeruginosa*	AHL/PQS	Virulence, biofilm formation, swarming, and exopolysaccharide	(28)
*Streptococcus pneumoniae*	AIP	Virulence, biofilm formation, and exopolysaccharide	(42)
*Lactiplantibacillus plantarum*	Plantaricin	Bacteriocin production	(39)
Fungi			
*Saccharomyces cerevisiae*	2-Phenylethanol, tryptophan, tyrosol	Drive filamentation and cell apoptosis	(28)
*Candida albicans*	Farnesol, tyrosol	Morphological changes, biofilm development, mating, drug efflux, and cell apoptosis	(40)
*Aspergillus nidulans*	γ-heptalactone, oxylipin	Growth and production of secondary metabolites	(43)
*Penicillium sclerotiorum*	γ-butyrolactone, polyacid	Production and metabolism	(43)
*Cryptococcus neoformans*	Pantothenic acid, Qsp1	Regulating virulence and host adaptation	(43)
*Ophiostoma floccosum*	Cyclic sesquiterpenes	Morphological changes, biofilm development, and drug efflux	(43)
*Aspergillus terreus*	Butyrolactone I	Growth phase-specific inducer and auto-stimulatory	(43)

^*a*^ CSP, competence-stimulating peptide; DSF, diffusible signal factor (a group of unsaturated fatty acids including cis-2-dodecenoic acid).

### Bacterial QS systems

Bacteria coordinate crucial biological functions through different QS systems, including virulence factor production (12), antibiotic biosynthesis (44), bioluminescence (45), and biofilm formation (10). Bacteria produce, release, and sense extracellular small molecules known as autoinducers (AIs). These molecules encompass diverse chemical classes depending on the bacterial species: AHLs are the primary signals in gram-negative bacteria, whereas AIPs are the predominant signaling molecules in gram-positive bacteria. When the concentration of AIs reaches a threshold level, the bacteria detect them and alter their gene expression (27). The QS system of *Escherichia coli*, a typical representative of gram-negative bacteria, includes the *LuxI/R*, the *LuxS* (synthase)/AI-2 (46), and AI-3 (47). Among these, there is a special class of QS regulatory proteins called SdiA (48). Termed an “orphan receptor,” SdiA lacks a cognate synthase but is nonetheless responsive to acyl-homoserine lactones (AHLs) produced by other bacteria. SdiA also acted as a transcriptional activator of the *ftsQAZ* operon (49), which encoded proteins essential for cell division. AIPs represent another important communication system mediated by gram-positive bacteria, and the most typical example is the *agr* system (50) in *Staphylococcus aureus*. When the extracellular AIP concentration reaches a threshold, it binds to the membrane receptor kinase AgrC, which, in turn, phosphorylates the regulatory factor AgrD. AgrD then cooperates with SarA to promote downstream expression of the *agrBDCA* genes, thereby regulating biofilm formation and virulence factor expression (51). Thus, in a cell density-dependent manner, bacteria employ diverse QSMs to regulate associated phenotypes and life processes (Table 1).

### Fungal QS systems

While research on bacterial QS systems in bacteria is relatively advanced, the understanding of fungal QS mechanisms, particularly in filamentous and pathogenic fungi, remains in its infancy. For these fungi, the identities of signaling molecules, their modes of action, and the full spectrum of regulated phenotypes are still poorly characterized. In recent years, growing attention has been devoted to exploring the roles of fungi in disease treatment and in the mechanisms underlying fungal infections (52). Within this context, an increasing number of studies have focused on the important regulatory functions of QS mechanisms in fungal biology.

Following the confirmation by Lingappa et al. (53) that farnesol is the first QS molecule identified in *Candida albicans*, subsequent studies have revealed the involvement of a growing number of terpenoids, alcohols, and peptide signaling molecules in fungal QS systems. *Saccharomyces cerevisiae* is the most well-known fungus and a common model for studying the role of QS systems in fungi. Chen and Fink (54) identified two aromatic alcohols, phenylethanol (PheOH) and tryptophol (TrpOH), as active QSMs that trigger filamentation. This conclusion was drawn by observing that cell culture supernatants of *S. cerevisiae* with a fixed growth cycle induced filamentation. The QS signaling molecules reported in *S. cerevisiae* include 2-phenylethanol (2-PE), tryptophol, and tyrosol. Lorenz et al. (55) also demonstrated that several alcohol products of amino acid metabolism, collectively referred to as fusel alcohols (e.g., 1-butanol), promote filament formation.

The morphological transition of *C. albicans* is regulated by a variety of environmental factors, among which QSMs play a critical role. *C. albicans* produces farnesol, which not only regulates its own phenotype but also affects the growth and QS behavior of other fungi. Notably, it exhibits tolerance to high farnesol, which is supplemented, which is lethal to other microorganisms (56). When supplemented with exogenous farnesol or secreted by *C. albicans* in biofilms, *S. aureus* exhibits significantly enhanced tolerance to antimicrobial agents (57), suggesting a potential interspecies QS crosstalk.

Filamentous fungi primarily utilize polyphenolic and lactone compounds as QSMs to coordinate developmental processes and the production of secondary metabolites. These fungi primarily exist as mycelia and spores. Their QS systems function to monitor population density and orchestrate key developmental processes, including sporulation, mycelial expansion, and the production of secondary metabolites such as antibiotics and toxins. *Aspergillus nidulans* and *Penicillium sclerotiorum* are typical models for studying QS mechanisms among filamentous fungi. However, research on the QS mechanism of *P. sclerotiorum* is relatively limited. However, existing evidence has shown that its QS system can significantly increase sclerotiorin production, demonstrating the important role of QS in the regulation of secondary metabolism. Pathogenic fungi rely more heavily on lipid and polypeptide signaling molecules to regulate virulence expression and host adaptability. Taking *Cryptococcus neoformans* as an example, the cell density-dependent growth behavior of this fungus is regulated by peptide molecules, showing similar characteristics to the bacterial QS system. *C. neoformans* can produce and secrete various QSMs, of which pantothenic acid (58) (PA) and the QS peptide Qsp1 (59) have been identified as key regulators of its pathogenic lifestyle (more details listed in Table 1, down). Table 1 reflects distinct ecological strategies: bacterial AHLs favor rapid coordination and pH-dependent “timing” in fluid niches. Conversely, fungal aromatic alcohols prioritize structural stability and metabolic robustness to regulate long-term morphological transitions in soil, favoring niche persistence over signaling speed.

## ANALYSIS OF QS-BASED COMMUNICATIONS

From the QS perspective, it is expected to deepen the understanding of the competition and cooperation between bacteria and fungi in the same ecological niche. Notably, QSMs produced by both bacterial and fungal species have been shown to mediate cross-kingdom signaling, a phenomenon especially common within the mixed-species architecture of biofilms and metabolic complementation (60, 61, 43). This section will summarize and analyze the phenotypes and underlying mechanisms regulated by QS-based communications between bacteria and fungi.

### QS regulates the formation of bacterial-fungal biofilms

The collaboration of different QSMs produced by bacteria and fungi can affect the gene expression of both parties during biofilm co-assembly, thereby affecting growth, morphological changes, and functional outcomes. The response of fungi to bacterial QSMs is widespread, which is mainly manifested in two typical pathogenic microorganisms, *C. albicans* and *P. aeruginosa*. The 3-oxo-C_12_-HSL (62), secreted by *P. aeruginosa,* and farnesol (63), produced by *C. albicans,* both contain a 12-carbon skeleton. They can affect morphogenesis by inhibiting the cAMP/PKA pathway (64) of *C. albicans*. The interaction increases the complexity of infections, particularly in chronic diseases involving polymicrobial biofilms.

**Fig 1:**
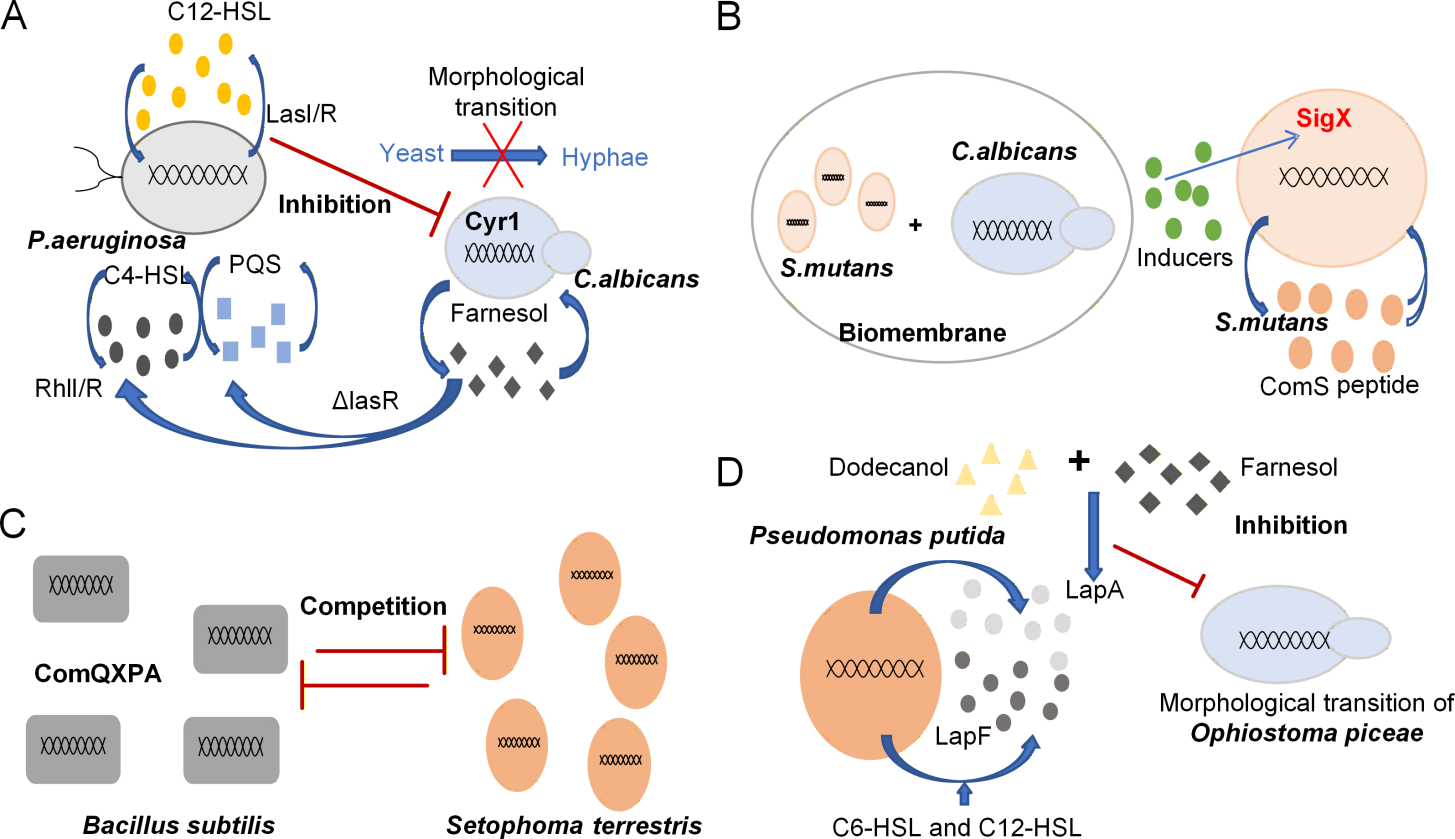
Four paradigms of bacterial-fungal interactions based on different QS systems. (**A**) *P. aeruginosa* can inhibit the morphological transition of *C. albicans* from yeast to hyphae. Meanwhile, farnesol, produced by *C. albicans*, can induce the production of C_4_-HSL and PQS in *P. aeruginosa* biofilm mutants. (**B**) A dual-species biofilm formed by *S. mutans* and *C. albicans* exhibits higher cell counts and biomass. (**C**) In the antagonistic interaction between the soil-borne bacterium *B. subtilis* and the fungal plant pathogen *S. scabies*, heritable phenotypic variation in the bacterium is mediated by mutations in the *ComQXPA* QS system. (**D**) *P. putida* KT2440 secretes two extracellular adhesive proteins, *LapA* and *LapF,* to form a biofilm. The dimorphic fungus *O. piceae* uses farnesol as a QSM to regulate its morphological transition from yeast to hyphal form.

Specifically, the 3-oxo-C_12_-HSL of *P. aeruginosa* can inhibit the adenylyl cyclase Cyr1 of *C. albicans*, thereby preventing the yeast-to-hyphae morphological transition. In contrast, the QSM farnesol of *C. albicans* can induce the production of QSM C4-homoserine lactone (C4-HSL) and quinolone signal PQS in the biofilm of *P. aeruginosa* lasR mutants (43), demonstrating a bidirectional regulatory effect (Fig. 1A). Similarly, in the co-cultivation system of *Streptococcus mutans* and *C. albicans*, the yeast-mediated bacteriocin *ComS* induces the expression of *sigX* and other competence genes (Fig. 1B). Compared with single-species biofilms, the activation of various QS systems in dual-species biofilms results in higher biomass, enhanced pathogenic potential, and reduced extracellular polysaccharides (65). The interaction between *C. albicans* and *S. aureus* is mediated by QSMs. The formation of antimicrobial-resistant biofilms affects various diseases, such as oral and intra-abdominal infections (66). Shi et al. (67) investigated the early stage of biofilm formation by different organisms in sewage. AHLs facilitate the synthesis of extracellular polymeric substances. Subsequently, tyrosol stimulates yeast cells to form germ tubes, which further promotes hyphal growth. In the later stage, these two types of signaling molecules synergistically enhance biofilm maturation and stability, generating a positive feedback effect on the biodegradation of various pollutants. These examples illustrate the clinical and ecological significance of bacterial-fungal interactions, particularly within the human host. A better understanding of the mechanisms underlying small signal molecules and bacterial-fungal communication mediated by these interactions can improve healthcare and disease control (68).

### QS regulates bacterial-fungal metabolic complementarity

QSMs produced by bacteria can affect the gene expression of fungi in the context of metabolic complementation, thereby modulating the dynamics of bacterial-fungal symbiosis. Similarly, fungal metabolites can either promote or inhibit bacterial growth. The interaction between the two can facilitate coevolution, which, in turn, affects the development of species in the environment. Andrea G et al. (69) investigated the strategies adopted by soil-dwelling *Bacillus subtilis* in its antagonistic interaction with *Setophoma terrestris* (ST) (Fig. 1C). The heritable phenotypic variations of the bacteria are mediated by mutations in the *ComQXPA* QS system, enabling the bacteria to gain an advantage in the antagonistic interaction. Ren et al. (70) found that C_12_-HSL and C_6_-HSL, two types of QSMs derived from bacteria, can alter the morphology of *S. cerevisiae* and affect its ethanol tolerance. AHLs can increase the frequency of cells with bipolar and multipolar buds. Meanwhile, long-chain HSLs slightly enhance the ethanol tolerance in *S. cerevisiae*, whereas short-chain HSLs significantly reduce it, suggesting that the regulatory impact of bacterial QSMs on *fungi* may be related to fungal growth pathways, as well as the morphological regulation and resistance of yeast to ethanol.

QSMs not only serve as communication mediators between bacteria and fungi but also induce extensive physiological responses in eukaryotic host cells. Farnesol can elicit multiple physiological effects in *S. cerevisiae* and *C. albicans*, including biofilm formation and oxidative stress. Alberto Ruiz et al. (71) evaluated the effects of signaling molecules on biofilm formation and structure during the co-cultivation of the fungus *Ophiostoma piceae* and *Pseudomonas putida* KT2440 (Fig. 1D). These molecules play a crucial role in microbial competition/cooperation and communication, with the most notable differences observed between fungi and bacteria. For instance, in the wheat rhizosphere, fusaric acid produced by the fungus *Fusarium oxysporum* inhibits the expression of genes encoding the antifungal metabolite 2,4-diacetylphloroglucinol (DAPG) in *Pseudomonas fluorescens* (72). In cystic fibrosis (CF) lung infections, the colonization of the co-fungal pathogen *Aspergillus fumigatus* and the opportunistic bacterium *Pseudomonas aeruginosa* increases expression of *lasB* in cells, thereby enhancing elastin production in *P. aeruginosa* (66). Bacteria and fungi synthesize various small signaling molecules to promote the growth of symbionts, inhibit antagonists, and thereby benefit their own community while defending against harmful competitors. For example, the mycorrhiza helper *Streptomyces* AcH 505 (44) produces a fungal growth-stimulating compound and fungal inhibitory molecules (WS-5995 B and C-antibiotics), thereby improving the hyphal growth of ectomycorrhizal fungi.

QSM can promote the evolution of coexistence among bacteria and fungi in a competitive environment. Jake N. Barber et al. (73) employed experimental co-cultures of *E. coli* and *S. cerevisiae* to investigate the evolutionary dynamics of species coexistence under resource competition. Although *E. coli* usually outcompetes *S. cerevisiae* in co-cultures, stable coexistence was observed in a small number of *S. cerevisiae* populations after approximately 1,000 generations of evolution. Scarinci et al. (74) used cross-feeding cocultures of *E. coli* and *S. cerevisiae* to demonstrate that, in a mutualistic community environment, direct physical contact and partner movement can provide fitness benefits to one or both parties. These benefits outweigh the costs incurred in co-cultures with low initial density and in the presence of non-cooperative counterparts.

To sum up, different bacterial and fungal populations use QS signaling molecules that are closely interconnected. The coordinated regulation of these signaling languages, in conjunction with the external environment, plays a crucial role in potential interactions between species and in cross-kingdom interactions with other organisms (43). In addition to the antagonistic and symbiotic interactions mediated by QSM, there are some other chemical signal-based communications between bacteria and fungi, such as volatile organic compounds (VOCs). Some VOCs exhibit density-dependent behavior similar to QS; they represent a unique communication modality due to their volatility and ability to cross phase boundaries. This regulatory process is particularly integral to the dynamics of pathogenicity in plant health and human diseases, such as lung infections, and plays a crucial role in the bidirectional communication between bacteria and fungi. They can also affect the gene expression of fungi, particularly in terms of coordinating virulence factors. VOCs can drive interspecific antagonism through their growth-inhibiting activity. For example, *Fusarium culmorum* releases a mixture of terpenes, a type of VOC, to influence the responses of *Collimonas pratensis* Ter 291 and *Serratia plymuthica* PRI-2C (75). Specifically, low concentrations of VOCs attract bacteria to fungi, while high concentrations of VOCs are toxic and can repel bacteria.

## ENGINEERING OF QS-BASED COMMUNICATIONS

Naturally occurring QS-based interactions are not invariably static; specific applications often demand highly precise and targeted regulation. Currently, the limited repertoire of endogenous promoter-allosteric transcription factor pairs presents a significant challenge in satisfying the concurrent demands for high-level expression (76), high sensitivity, and application versatility (77). Consequently, acquiring a library of high-performance regulatory components through synthetic engineering is critical for achieving precise expression of pivotal target genes and modulating bacterial-fungal interactions. Engineering promoter-transcription factor combinations (78) is frequently essential to enable controllable transcriptional tuning, including fine adjustments to expression intensity. This is typically pursued via two complementary strategies: the rational redesign of core promoter elements (79) and screening of element libraries using directed evolution methods (80).

### Promoter engineering

Due to the structural and functional differences between prokaryotic and eukaryotic promoters, strategies must be adapted according to the host type. Prokaryotic promoters typically exhibit a well-defined architecture, with two highly conserved elements located at approximately −10 and −35 positions. For most prokaryotes, the consensus sequences of the −10 and −35 regions are TATAAT and TTGACA (81), respectively, with a non-conserved nucleotide spacer in between. The transcriptional activity of prokaryotic promoters may be affected by any of these elements, making them amenable to promoter engineering for tunable gene expression. In contrast, eukaryotic promoters are more complex and modular, consisting of a core promoter region and upstream activating sequences (UAS). The common strategies for promoter engineering for these two types of promoters currently include two approaches: one is to perform mutagenesis on endogenous promoters to enable binding to specific transcription factors and thereby alter their regulatory properties, and the other is to replace the original promoter with promoters from different sources for combinatorial optimization, which can change the gene expression profile and achieve artificial control at the transcriptional level.

**Fig 2:**
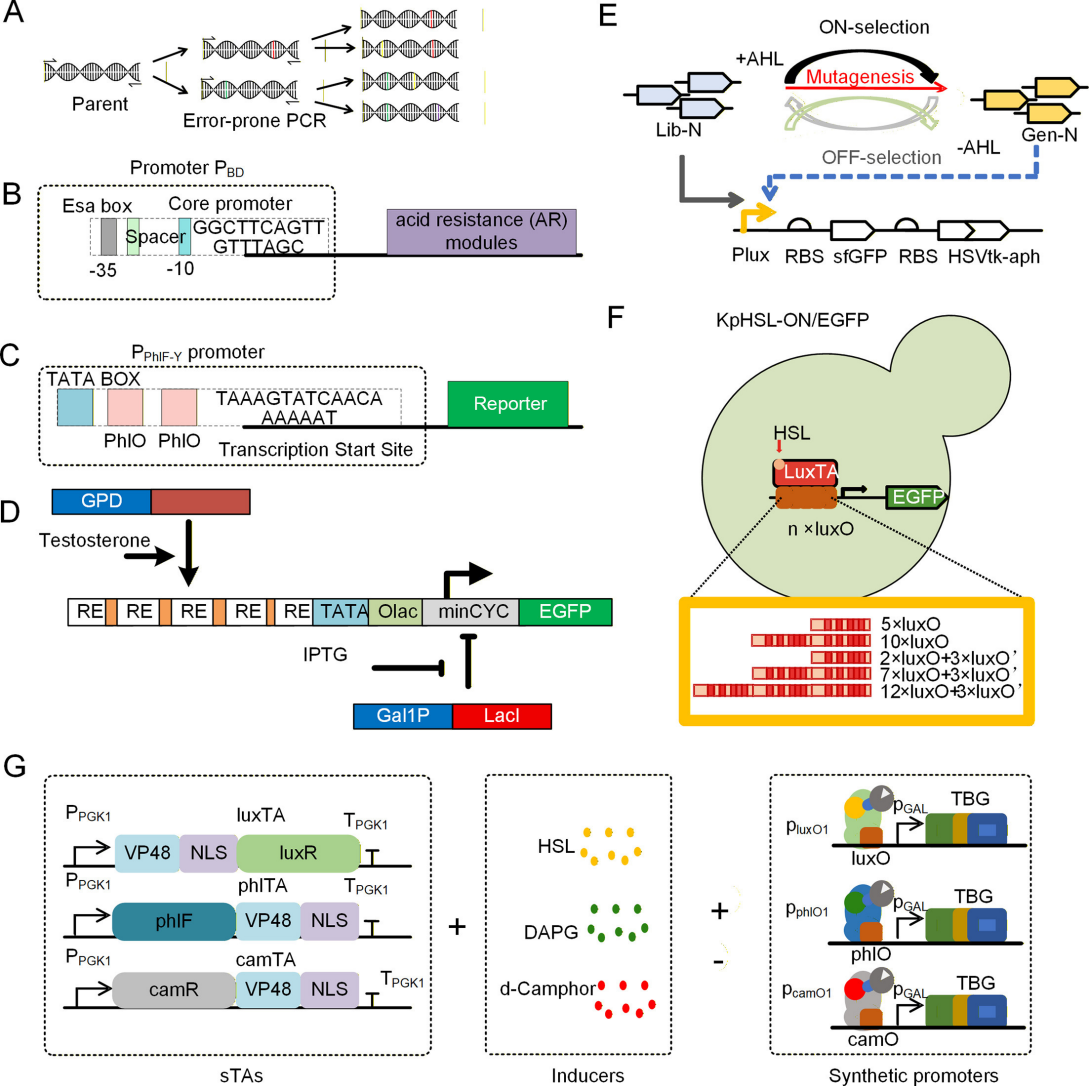
Optimization design and directed evolution methods for QS elements. (**A**) Random mutagenesis, such as error-prone PCR (epPCR), which introduces point mutations that mimic the imperfect DNA replication process, allows for the generation of diverse promoter variants. (**B**) A schematic diagram of assembling the QS P_esaS_ promoter mutant for regulating the acid-tolerant module. (**C**) Integration of the bacterial phlF operator with the yeast P_gal1_ promoter to create a synthetic, orthogonal promoter for *de novo* designed intercellular communication systems. (**D**) Based on the core part of the minimal cytochrome C (minCYC) promoter, five steroid hormone response elements (SHRE) were added upstream and downstream of the TATA box, and a lac operator site was also included. This configuration allows for precise dual-control, where SHRE facilitates testosterone-dependent activation and the lac operator enables IPTG-mediated inhibition. (**E**) Directed evolution for the stringency of the LuxR/P_lux_ inducible system. The goal was to enhance system stringency by minimizing "leaky" expression in the absence of the AHL ligand through multiple rounds of mutagenesis and selection. (**F**) A schematic diagram of iSynP in the inducible system responsive to HSL. An HSL-inducible system where the repeat number of bacterial operators is optimized to achieve robust and tunable signaling responsiveness. (**G**) Plasmid-based expression of the identified sTA variants provided dose-dependent activation of genes placed under the control of *p_phlO1_*, *p_camO1_*, or *p_luxO1_* in host cells.

In terms of promoter mutation, random mutation (e.g., error-prone PCR and synthetic promoter library construction) and site-directed mutation (e.g., saturation mutation and domain replacement) are two commonly used methods. Through these methods, researchers can construct promoter libraries with different expression strengths, known as Synthetic Promoter Libraries (SPL), which are applied in metabolic engineering for the differential expression regulation of multiple genes. Subsequently, combined with high-throughput screening technology, promoters with excellent performance can be rapidly screened out (82) (Fig. 2A). The advantage of random mutation lies in its ability to generate a broad promoter library with a gradient of expression strengths, thereby providing abundant options for fine-tuning gene expression. However, this method usually requires extensive screening work and relies on an efficient screening platform. A modular QS switch (83) (*PhrC-RapC-SinR*) has been developed in *B. subtilis*, which can be used for the dynamic and fine regulation of the side pathway for menaquinone-7 (MK-7) synthesis, thus significantly increasing the yield of MK-7. This QS system has been successfully integrated with biocatalytic functions. It has potential applicability for fine-tuning gene expression in a wide range of microorganisms, as well as enhancing the production of metabolites. In contrast, the *Esa* QS circuit can dynamically regulate the expression of the AR module (*DsrA-Hfq*). Through the elaborate design and stepwise evaluation of the key promoter *P_esaS_* (Fig. 2B), an optimal *Esa-PBD* (L) circuit (84) has been developed. This circuit enhances the productivity and yield of amino acids in the industrial *Escherichia coli* strain SCEcL3 under pH 5.5 conditions. Ge et al. (19) employed promoter engineering to optimize the core regulatory elements of the *lux*-type QS system. By precisely tuning the number of CRP-binding sites and redesigning the lux box sequences, they developed a library of QS variants characterized by high dynamic ranges and low basal leakiness, ultimately achieving an 11.3-fold increase in 4-hydroxycoumarin production.

Promoter compatibility and functionality vary significantly across diverse host systems. The development of broad-spectrum synthetic promoters suitable for multiple hosts has become a crucial direction in expanding the synthetic biology toolkit. Due to variations in genetic backgrounds and transcriptional mechanisms among different strain types, significant disparities in the expression efficiency of a given gene across diverse hosts can result. Such promoters characteristically integrate fundamental yet adaptable promoter elements from different species, thereby attaining stable and efficient expression of downstream genes in heterologous hosts. For instance, the introduction of heterologous regulatory elements (e.g., the bacterial tetracycline operator *tetO* and the lactose repressor *LacI*) through engineering design has been shown to significantly enhance the performance of biosensors without interfering with the host organism’s original regulatory network.

To ensure the reliability of engineered cross-kingdom communication, it is imperative to adopt rigorous quantitative metrics for orthogonality. A central standard for assessing cross-kingdom signaling orthogonality is to maintain cross-activation at a level comparable to basal expression (85), while utilizing response matrices, dynamic range, and signal-to-noise (S/N) ratio as complementary metrics to rigorously quantify the independence and functional robustness of these communication channels. Du et al. (86) designed two novel DAPG-PhlF Sal-NahR communication channels and conducted rational design on six commonly used QS systems, thereby obtaining an orthogonal regulatory system capable of bidirectional communication in *E. coli* and *S. cerevisiae*. As shown in Fig. 2C, by inserting two PhlF operators between the TATA box and the transcription start site of the P_Gal1_ promoter, it was achieved that the DAPG synthesized by *E. coli* could be detected in *S. cerevisiae* cells. The integration of multiple *tetO* copies with the yeast CYC1 promoter facilitates the construction of hybrid promoters (Fig. 2D), which exhibit controllable expression characteristics in response to varying tetracycline concentrations. The expression intensity of these hybrid promoters is comparable to that of the classical *P_GAL1_* promoter (87). By employing rational design and directed evolution methodologies, a biochemical channel toolkit can be engineered for multi-channel communication in pattern formation and distributed cellular biological computing applications. The redesign of channels suitable for mammalian systems, in conjunction with the optimization of the receiver module through directed evolution, has been demonstrated to yield enhanced dynamic range and sensitivity. In turn, this has been shown to reduce the metabolic burden on sender cells and improve the channel compatibility of eukaryotic receiver cells.

In order to enhance the robustness of biosynthetic pathways, the construction of feedback regulatory networks that respond to metabolic intermediates or environmental stressors has emerged as a pivotal approach for precise gene expression control. In practical applications, a single stimulus-response may be insufficient to achieve precise regulation (88) of target genes, and promoter engineering can be employed to optimize the sensitivity and dynamic response range of biosensors. By combining multiple regulatory elements, it is possible to regulate multiple signals, thereby expanding the response dimensions of fungal biosensors. For example, placing multiple androgen receptor binding sites upstream of the *CYC1* promoter and combining a *LacI* binding site downstream creates a promoter system with a broad activity range under dual-signal regulation by testosterone and isopropyl β-D-thiogalactoside (IPTG) (87). Michael et al. (89) discovered that the *Agr* system in *S. aureus* consists of the synthetase *AgrD*, which produces the initial peptide ProAIP. This peptide is then cyclized by the membrane protein AgrB and exported outside the cell. Once outside the cell, the signal peptide is cleaved by the peptidase SpsB to form the mature 8-amino acid AIP-1. This mature signal can then be recognized by the membrane-bound histidine kinase AgrC, which phosphorylates the cytoplasmic regulator AgrA and simultaneously activates the cognate promoter to regulate the target function. Combining promoters with different functions enables the construction of multi-layered regulatory networks, allowing more complex control of gene expression. In fungi, QS mechanisms often utilize pheromone-responsive promoters (e.g., *FUS1*) to sense cell density or population status. Meanwhile, the *ARO9* promoter can respond to aromatic amino acids (90) in the culture medium and drive pheromone expression. Therefore, QS of pheromones can be dynamically regulated by changes in the concentration of aromatic amino acids, creating a feedback regulatory mechanism based on environmental signals. The number and position of exogenous regulatory DNA sequences affect the induction rate and dose-response curve of biosensors, making their design crucial for creating sensitive and robust biosensors. Successful application of heterologous elements in promoters endows biosensors with new properties, enabling them to respond to additional metabolites or chemicals and facilitating their use in precisely regulating gene expression in biosynthetic pathways.

In summary, although the QS mechanism mediated by AHLs has been extensively studied in bacteria, significant differences exist in the types of signaling molecules and regulatory mechanisms found in fungi. Nevertheless, existing studies have demonstrated that the precise regulation of fungal population behavior can be achieved by modifying and optimizing regulatory elements (e.g., promoters, transcription factors, and biosensors). These studies contribute to our understanding of the molecular mechanisms underlying QS in fungi and provide theoretical and technical support for constructing fungal cell factories with adaptive capabilities.

### Targeted evolutionary modification of QS regulatory elements

Bacteria contain a plethora of diverse transcriptional regulatory elements (91, 92), among which regulators of the LuxR family have been extensively optimized and applied in transcriptionally activating elements. However, fungi are deficient in possessing diverse and high-performance genetic switches. Although QS regulatory elements (93) usable in heterologous species have been obtained through rational design and screening in promoter engineering, they still cannot meet the needs of synthetic modification of microecosystems. Consequently, recent studies have sought to identify *luxR* QS regulatory elements that can be employed in fungi through the utilization of directed evolution methodologies. The objective of directed evolution is to accelerate the natural evolutionary process of biomolecules and systems *in vitro* through repeated rounds of mutagenesis and high-throughput screening/selection (82). Due to its high success rate, rapidity, and comprehensiveness, directed evolution has been widely applied in protein evolution, metabolic pathway optimization, and even the design and improvement of entire genomes in the pharmaceutical field.

In the development of universal genetic switches within co-culture systems, this article primarily summarizes the latest advancements and modification strategies for applying metabolite-responsive transcriptional regulatory elements derived from bacteria to the development of yeast genetic switches. Yuki et al. (40) obtained a stringent LuxR receptor protein through four consecutive rounds of ON/OFF screening. This was achieved using the *hsvTK*-aph-sfgfp module-based screening (Fig. 2E), where the fusion of aph kanamycin kinase and fused *hsvTK* (herpes simplex virus thymidine kinase) enabled simultaneous positive and negative screening. The screened mutant ligands exhibited low leaky expression in the absence of signal molecules, thereby significantly enhancing the signal-to-noise (S/N) ratio of the QS system. Similarly, an additional study initially generated a substantial library of mutants for the regulators *NahR* and *PhlF* via error-prone PCR. This was followed by multiple rounds of negative and positive screening to obtain optimized elements with considerably enhanced sensitivity and dynamic range. These two communication elements were subsequently employed to achieve bidirectional communication between *S. cerevisiae* and *E. coli* (86).

In order to (94) develop strong inducible synthetic promoters with minimal leakage (Fig. 2F), we significantly enhanced the regulatory capacity of the promoters through the insertion of insulator sequences and operator optimization. The construction of inducible synthetic promoters (iSynPs) has been achieved, thus enabling the expansion of the yeast promoter library with the desired characteristics. The universal design and applicable method for constructing tightly regulatable iSynPs in yeast include the following: the insertion of insulator sequences larger than 1 kbp to prevent transcriptional activation from upstream cryptic activating sequences; the direct fusion of operators upstream of the TATA box; and the increase in the number of operator repeats or the screening of (mutation) bacterial operators to reduce their cryptic activation without affecting the binding to synthetic transcriptional activations (sTAs). Despite the non-monotonic effect of the number of operator sequences on induction, an increase in the number of operator repeats has been shown to enhance the induction fold of the system monotonically. Tominaga et al. (95) constructed a platform for the rapid evolution of yeast genetic switches, utilizing a trifunctional fusion protein *hsvTK* -Ble-GFP (TBG) (Fig. 2G). Utilizing the stability and flexibility of this directed evolution platform, they engineered a bacterial-derived Lux QS regulatory pathway to achieve efficient activation in *S. cerevisiae* cells; in comparison with the original elements, both the S/N ratio and sensitivity were significantly improved (40). The resulting genetic switches can be readily integrated into biosynthetic pathways to achieve selective AND-gated control, thus facilitating the reconstruction and implementation of various yeast genetic switches for diverse applications. While promoter engineering and directed evolution are foundational for creating specificity, Wu et al. (96) have further detailed strategies utilizing metabolites, RNA, and orthogonal translation to ensure that these synthetic circuits do not interfere with the host’s intrinsic mechanisms. These approaches are critical for achieving true orthogonality, where the synthetic device and the host cell operate as functionally independent modules.

To sum up, the interactions between bacteria and fungi are widespread in nature, influencing the behaviors and functions of both organisms to varying degrees through mechanisms such as signal transduction, material exchange, and metabolic regulation. These interaction mechanisms are of great significance in basic biological research, as well as providing theoretical support and technical approaches for the optimization of industrial fermentation, the development of biological control strategies, and the design of novel microbial agents.

## CONCLUDING REMARKS AND FUTURE PERSPECTIVES

Microorganisms produce different types of small signal molecules (97, 98), which collectively serve the common goals of self-defense and interspecific communication. QSMs play a crucial role in the ecological interactions between bacteria and fungi, not only determining interspecific coevolution and environmental adaptation but also offering potential applications in biofilm formation (99) and disease prevention (100). An in-depth analysis of the signal transduction mechanisms of QSMs and their functions in various environments will facilitate the development of novel microbial regulation strategies (101), providing theoretical support and technical references for fields such as ecological control (102), plant health (103), and environmental restoration (104).

Although the research on the QS system in the filamentous fungi is still in the exploratory stage (105), it has shown great potential in regulating key behaviors during the fungal life cycle (106), such as sporulation, morphogenesis (107), and secondary metabolite production (108). However, compared with the QS research system in bacteria, the current understanding of the QS mechanism in filamentous fungi remains relatively limited. This is particularly true for aspects such as the recognition mechanism of signal molecules, the structural and functional analysis of receptor proteins, and the elucidation of signal transduction pathways, which all require further in-depth investigation to fully understand their regulatory roles and potential applications.

Note that the combination of directed evolution with multi-species interaction research is expected to further advance synthetic biology towards more complex and precise regulatory systems, thus bringing revolutionary progress to fields such as biomanufacturing, medicine, and environmental governance (109). In recent years, machine learning has demonstrated considerable potential in the field of synthetic promoter design (110). Conventional promoter design is predicated on the iterative process of experimentation and the accumulation of experience (111). In contrast, machine learning models have the capacity to predict the activity of synthetic promoters on the basis of large-scale gene expression data and sequence features and to generate sequences that meet design objectives rapidly. This approach has been demonstrated to enhance design efficiency to a considerable degree (112), while concomitantly facilitating a more profound comprehension of the gene expression regulatory mechanisms operating at the level of the entire genome. Furthermore, the interaction network formed by bacteria and fungi through QSMs and metabolites is a crucial driving force in their coevolution (113). It also provides a theoretical basis for the further in-depth exploration of the molecular mechanisms underlying bacterial-fungal interactions, the identification of more key signaling molecules and regulatory genes, the development of new antibacterial agents, and the optimization of environmental bioremediation technologies.

As core microbial components in natural ecosystems, the interaction between bacteria and fungi constitutes a key link driving the stability of community structure and material cycling. Within soil communities, bacteria and fungi facilitate carbon and nitrogen cycling (82) through metabolite exchange, thereby enhancing soil fertility (58). In plant symbiotic systems, mycorrhizal fungi and plant growth-promoting rhizobacteria (PGPR) work synergistically to strengthen plant stress resistance (114). In gut microbiota, they maintain host health (6) through complex interaction patterns. Therefore, an in-depth analysis of bacterial-fungal interaction patterns in various natural environments not only reveals the intrinsic mechanisms of ecosystem functioning but also provides a scientific basis for soil remediation and biological control, holding significant implications for maintaining ecological balance and promoting sustainable development.

With the continuous advancement of bioinformatics tools and artificial intelligence technologies, the systematic mining and functional prediction of QS elements and quorum-sensing communication networks (QSCN) have become more efficient and accurate (115). Specifically, AlphaFold2 (116) predicts single-pass receptor structures for precision engineering, and Bayesian optimization (117) streamlines the tuning of complex QS genetic circuits. Potential signaling molecules, regulatory elements, and their interaction networks are identified from high-quality genomic data, thereby accelerating the design and optimization of artificial QS systems. For instance, predicting the response relationships between different QSMs and promoters through machine learning models can significantly shorten the screening cycle and improve the success rate of design. Meanwhile, the in-depth understanding of the modular organization mechanism of QS signaling networks in prokaryotic and eukaryotic cells enables synthetic biologists to draw on their structural characteristics to design engineered cells with novel signaling behaviors. For example, by introducing QS modules from bacteria into yeast, cross-species genetic switches can be constructed to achieve precise control of specific behaviors in multicellular systems. This thus translates mechanistic insights into direct ecological interactions between fungi and bacteria, yielding ecological interventions that benefit human health and development.

Nevertheless, there are several technical challenges that still need to be addressed. Among these, cross-talk between multi-signal networks is a key issue that limits system performance, particularly in complex co-culture systems where different signaling pathways may interact, leading to regulatory inaccuracies. In addition, the utilization of real-time quantitative monitoring methods for QS signals *in vivo* remains in its infancy, thus impeding the real-time analysis and regulation of signal transmission processes. Consequently, future research should concentrate on the orthogonal execution of multi-signal systems, high-sensitivity *in vivo* signal detection technologies, and the optimization of cross-species QS compatibility.

Apart from QSM, VOCs also play a pivotal role in mediating the two-way communication and competition (118) between bacteria and fungi, functioning through a chemical signaling mechanism akin to quorum sensing (119). VOC-mediated microbial communication can affect the pathogenesis of diseases: *P. aeruginosa* produces volatile substances (dimethyl sulfide), which stimulate the growth of *Aspergillus fumigatus* in the initial stage of CF lung infection. However, when in direct contact, these two microorganisms antagonize each other’s growth, competing for nutrients. Similarly, *A. fumigatus* in CF airways inhibits *P. aeruginosa* by producing gliotoxin, which significantly affects the composition of the airway microbiota (120).

To sum up, QS is the core language of bacterial-fungal cross-kingdom interactions, and it holds significant value in both fundamental research and practical application. Precise regulations of QS signal generation and response enable targeted guidance of microbial community behaviors. Furthermore, QS-based ecological interactions between bacteria and fungi reveal both the complexity of microbial communication in nature and the immense potential for artificially designing QS systems in synthetic biology. Beyond biomanufacturing, QS modulation offers transformative clinical potential. In anti-infective therapy, quorum quenching can disrupt bacterial communication and biofilm formation to attenuate virulence without antibiotic resistance. Additionally, integrating QS circuits into probiotic chassis enables “smart” bacteria to sense disease biomarkers and trigger localized drug delivery, establishing a new paradigm for precision microbiome-based treatments. Ensuring cross-kingdom biosafety is critical. A multi-layered biocontainment strategy was established by integrating metabolic auxotrophy and programmed kill switches (121). Obligatory dependence—engineered via metabolic cross-feeding or non-canonical amino acids—strictly constrained the synthetic consortium. Furthermore, active CRISPR-Cas or toxin-antitoxin switches ensured cell death in response to environmental triggers. These synergistic safeguards significantly mitigate risks of horizontal gene transfer and unintended environmental escape. By thoroughly analyzing the regulatory mechanisms of QS in natural interactions and utilizing bioinformatics and artificial intelligence technologies, researchers can more efficiently identify and optimize QS components. This paves the way for applying these bacterial-fungal QS communication networks across a diverse range of fields, including anti-infective therapy, sustainable agriculture, and biomanufacturing.
